# Deciphering Carotenoid and Flowering Pathway Gene Variations in Eastern and Western Carrots (*Daucus carota* L.)

**DOI:** 10.3390/genes15111462

**Published:** 2024-11-13

**Authors:** Sarvamangala S. Cholin, Chaitra C. Kulkarni, Dariusz Grzebelus, Rashmi Jakaraddi, Aishwarya Hundekar, B. M. Chandan, T. S. Archana, Nair R. Krishnaja, G. Prabhuling, Gabrijel Ondrasek, Philipp Simon

**Affiliations:** 1 Plant Molecular Biology Lab (DBT-BIO-CARe), Department of Biotechnology & Crop Improvement, College of Horticulture, University of Horticultural Sciences, Bagalkot 587103, Karnataka, India; kchaitra642@gmail.com (C.C.K.); rashmiijakaraddi@gmail.com (R.J.); haishwarya4@gmail.com (A.H.); chandanbm4@gmail.com (B.M.C.); archanasathyansath@gmail.com (T.S.A.); krishnaja1993@gmail.com (N.R.K.); gprabhuling@gmail.com (G.P.); 2 Department of Biotechnology & Crop Improvement, Kittur Rani Channamma College of Horticulture, Gokak 591218, Karnataka, India; 3 Department of Plant Biology & Biotechnology, University of Agriculture in Krakow, Al. Mickiewicza 21, 31-120 Krakow, Poland; d.grzebelus@urk.edu.pl; 4 Department of Soil Amelioration, Faculty of Agriculture, University of Zagreb, HR-10000 Zagreb, Croatia; gondrasek@agr.hr; 5 USDA-ARS Vegetable Crops Research Service, Madison, WI 53706, USA

**Keywords:** eastern carrot, western carrot, domestication, carotenoid, flowering genes

## Abstract

Background/Objectives: Carrot is a major root vegetable in the *Apiaceae* owing to its abundant carotenoids, antioxidants, vitamins, and minerals. The modern dark orange western carrot was derived from sequential domestication events from the white-rooted wild form to the pale orange-, purple-, or yellow-rooted eastern carrot. Genetic and molecular studies between eastern and western carrots are meager despite their evolutionary relatedness. Methods: Twelve RNA seq libraries obtained from distinct eastern and western cultivars at vegetative and reproductive developmental stages were utilized to identify differentially expressed genes (DEGs) to decode the key molecular genetic changes in carotenoid and flowering pathways. Results: In the carotenoid pathway, an upregulation of the PSY, CRTISO, and LCYE genes was observed in the western cultivar, while the eastern cultivar exhibited a higher abundance of downstream enzymes, particularly CCD and NCED1. These later enzymes are crucial in linking apocarotenoids and xanthin-mediated ABA signaling. In the flowering pathway, we noted a greater expression of DEGs associated with the photoperiod and vernalization pathways in the western cultivar. In contrast, the eastern cultivar displayed a dominance of genes from the autonomous pathway (FLD, LD, FLK, and PEBP) that function to repress FLC. The experimental validation of 12 key genes through quantitative real-time PCR further confirms their functional role in carrots. Conclusions: The identified key regulatory genes in these major pathways are valuable for designing breeding strategies for manipulating carotenoid content and flowering time while developing climate-specific carrots. The knowledge of carotenoid and flowering pathways is advantageous in producing nutritionally improved roots and seeds in carrots across diverse climates.

## 1. Introduction

Carrot (*Daucus carota* subsp. *sativus* L.) is a nutrient-rich and delicious root vegetable consumed by people of all age groups [1]. In terms of its economic value and nutritional benefits, carrot is considered as the second most important vegetable crop [2]. Due to its protandrous nature, carrot is a highly outcrossing crop, resulting in broader genetic diversity within the cultivated gene pool. Eastern (*D.c.* ssp. *sativus* var. *atrorubens Alef*.) and western (*D.c* ssp. *sativus* var. *sativus*) carrots are major cultivated and globally grown groups [3,4]. Both are diploid spp. with a chromosome number of 2*n* = 18 [5] and a genome size of 473 Mb [6,7]. A recently published high-quality genome was derived from a western, Nantes-type carrot with a genome coverage of 421.5 Mb [6] and 440.7 Mb [7], as well as a Kuroda-type carrot with a genome coverage of 430.40 Mb [8]. Cultivated western carrots result from two domestication events: initially from white-rooted wild forms to purple- or yellow-pigmented eastern carrots through primary domestication and further to carotene-rich dark orange western types through secondary domestication [9,10].

Both eastern and western cv types differ largely in secondary storage root morphology, viz., xylem–phloem patterning, root shape, texture, quality, vernalization requirements, flowering behavior, and growth habits [3,10,11]. Both cultivars differ in their ability to respond to varied agroecological conditions [9,10,12]. The genome resequencing of diverse *D. carota* subspecies phylogenetically grouped these two types into distinct clades with a clear population differentiation (Fst) based on nucleotide diversity [6,13]. Despite the wide range of carrot diversity, most achievements in carrot improvement, viz., the development of superior varieties/hybrids and genetic or transcriptomic investigations, have primarily focused on biennial western carrots adapted to temperate regions. Western carrots have acquired many desirable selection signatures, viz., high carotenoid content, nonbranching, uniform roots, an attractive shape, a smooth texture, the absence of hairs, and a biennial nature to avoid early bolting during the vegetative phase, though the domestication process [3,9,10]. In contrast, eastern carrots grown in China, India, Japan, and Afghanistan have an ununiform root shape and color, a coarse root texture, distinct xylem–phloem patterning, wider adaptations to varied environments, and an annual growth habit that does not require vernalization for floral induction [3,10,11].

Carrot breeders have made notable strides in recent decades, achieving a remarkable average increase in carotenoid content compared to levels from four decades ago [1,6]. As a result, highly valued modern varieties and hybrids like Imperator, baby carrots, Nantes, Kuruda, Chanteny, and Danvers were made available to consumers in temperate regions. Global carrot production has also significantly increased due to the availability of high-value carrots for fresh consumption and processing [1,6,14]. However, these modern and superior carrots are least utilized by the growers and consumers of sub/tropical nations like India, mostly due to limited seed availability, high seed costs, and challenges in seed production in warmer climates because of their obligate vernalization requirement.

The carotenoid pathway is the most studied n carrot and many other food crops owing to its nutritional value in maintaining eye health [15,16]. The carotenoid pathway not only produces nutritionally valuable α- and β-carotenes, lutein, or lycopene but also plays a major role in defense response by diverting to phytohormone pathways such as abscisic acid (ABA) or stringolactone [8,16]. Carrot is a model system for storage root development and carotenoid accumulation [6]. Eastern carrot cvs are mostly grown in the tropics lacks in carotenoid because high-carotenoid genes (*Y*, *Y2*, and *Or*) evolved during the post-secondary domestication period in the western carrot cvs [9]. Vitamin A deficiency, a devastating human disease-causing night blindness due to a shortage of vitamin A, is more frequent in tropical countries [17]. Vernalization has been an important domestication trait for temperate climates. Conversely, it poses a significant obstacle to the high-carotenoid western cultivar’s ability to produce seeds in tropical areas. To use western cvs in warmer regions, researchers must consider other endogenous routes that stimulate flowering, such as autonomous or hormone-regulated processes. Hence, deeper insights into two important traits such as the carotenoids and flowering behavior of eastern and western carrot cvs would be essential for developing nutritionally superior and climate-specific varieties.

Transcriptomics is the most powerful bottom-up genomic tool to investigate and elucidate the genetics of plant domestication [9]. Thus, to ascertain the likely secondary domestication changes in carotenoid and flowering pathway genes, transcriptome profiling was performed in two different carrot cultivars (cvs), viz., western (Kuroda-type) and eastern, using vegetative (storage root) and reproductive phase (flower primordia and leaf(FPL)) tissues. In addition, validation of candidate genes through quantitative real-time PCR (qRT-PCR), and key molecular changes in the carotenoid and flowering pathways are discussed below.

## 2. Material and Methods

### 2.1. Plant Material

**Figure 1:**
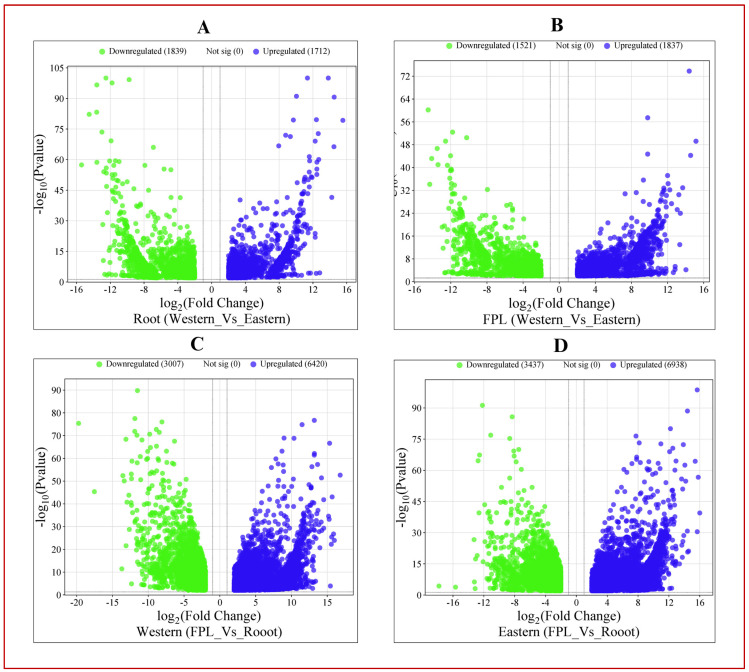
Pair-wise comparison of up- and downregulated DEGs across tissues and cultivars (*p* = 0.01 and Log2FC |±2|). (**A**) DEGs up- and downregulated in western and eastern cultivars, respectively, in root tissue. (**B**) DEGs up- and downregulated in western and eastern cultivars, respectively, in FPL tissue. (**C**) DEGs up- and downregulated in FPL and root tissue, respectively, in western cv. (**D**) DEGs up- and downregulated in FPL and root tissue, respectively, in eastern cv.

An annual-type eastern cv (*Daucus carota* ssp. *sativus* var. *atrorubens* Alef., UHSBC-23-1) and biennial-type western carrot (*Daucus carota* ssp. *sativus* var. *sativus;* UHSBC-100) were selected in this study (Figure 1). UHSBC-23-1, a red-colored carrot developed at the UHS Bagalkot belonging to the eastern type, with a higher root weight, TSS, and lesser β-carotene than the western dark orange carrot cultivar UHSBC-100. There is a significant morphological difference in root morphology, root quality, productivity, and flowering behavior among tested cvs in the vegetative [10] and reproductive phases. The experiment was conducted at the University of Horticultural Sciences Bagalkot. Bagalkot (16°12′ N, 75°45′ E) receives an average rainfall of ~318 mm and belongs to the northern dry zone of Karnataka. The test cvs were subjected to a vegetative phase experiment to produce healthy roots during rabi season in 2020 in the red sandy loam soil at the greenhouse facility [10]. The reproductive phase experiment was conducted in the phytotron facility by utilizing the shoulders of the roots of test cultivars. Flowering was induced by providing the critical day length (16 h light and 8 h dark) under far-red light in the cold room maintained at 4–8 °C. After the floral transition, floral primordia (minimum of 0.3 mm in size) with the attached leaf tissue hereafter mentioned as ‘FPL’ were utilized for RNAseq. After the vegetative phase, the shoulder portions of the carrots (stackings) were evaluated for their ability to induce flowering in cold room conditions and natural field conditions. The observations were recorded on important plant morphological parameters such as plant height (cm), the number of petioles, the length of the petiole (cm), petiole thickness (mm), the number of days to induce flowering, and bud thickness (cm) when the plants showed floral induction with a minimum of 3.00 mm bud thickness.

### 2.2. Selection of Tissues for RNA Seq

Two tissues, (i) a mature storage ‘root’ (sampled during the vegetative phase) and (ii) flower primordia with attached leaf, i.e., ‘FPL’ tissue (sampled during reproductive cycles), were used for RNAseq. The root was sampled at a fully matured marketable stage at 90 days after sowing (DAS), whereas FPL tissue was sampled when the floral primordia attained a size of 3 to 4 mm thickness. Each cultivar consisted of three biological replicates in each tissue were promptly frozen in liquid nitrogen. Cryopreserved samples were immediately delivered to Agrigenomics Pvt Ltd. Kochi, Kerala-682037, India in dry ice for RNA isolation and sequencing.

### 2.3. RNA Isolation, Library Preparation, and Sequencing

Isolated RNA was examined in Bioanalyzer. Samples with an RNA integrity number (RIN) > 8.0 were subjected to cDNA synthesis and library construction for RNA-seq as explained in [10]. Samples from both experiments were independently subjected to RNAseq analysis on different platforms such as Hiseq 4000 for root tissues with 2 × 100 nt paired-end (PE) libraries and Novaseq 6000 with 2 × 151 nt PE libraries for FPL tissues (Supplementary Table S1). The raw reads were preprocessed to ensure high-quality reads for de novo assembly construction. To achieve the highest read quality of Q > 30, the tools FastQC, developed by the Babraham Institute [18], trimmomatic v 0.36, developed by USADELLAB [19], and trimgalore, developed by the Babraham Institute [20], were used. The settings were established in trimmomatic (ILLUMINACLIP: TruSeq3-PE.fa:2:30:10:2:keepBothReads TRAILING:20 MINLEN:30 AVGQUAL:20) and trimgalore (max_n 2, paired, length 36) for root samples with 100 nt paired-end (PE) reads. For the RNAseq libraries of FPL tissue having 151 nt PE reads, the parameters were set in trimmomatic (ILLUMINACLIP: TruSeq3-PE.fa:2:30:10:2:keepBothReads TRAILING:20 MINLEN:50 AVGQUAL:20) and in trimgalore (max_n 2, paired, length 50). After the filtering and removal of duplicates and low-quality reads, a total of 374,175,674 reads were retained for de novo assembly construction (Supplementary Table S1).

### 2.4. De Novo Transcriptome Assembly, Quality Assessment, and Abundance Estimation of Transcripts

The de novo assembly was constructed from 12 RNAseq libraries using the Trinity v 2.8.6 pipeline [21], with the following parameters: a minimum contig length of 200 nt, minimum K-mer coverage of 30, minimum k-mer covariance of 2, minimum 98 percent identity, and maximum internal gap of 10. Several techniques were used to evaluate the assembly quality. To calculate the percentage of mapping of particular libraries, the processed RNA-seq reads were first mapped to their respective de novo transcriptome assembly (Supplementary Table S2). Further assembly was compared to a database of single-copy orthologous genes for plants that were built using BUSCO v.1.161 [22] with >90% completeness (Supplementary Figure S1). By using CD-HIT EST clustering with a 0.95 similarity, the duplicated transcripts were eliminated from the assembly. Finally, a high-quality assembly was generated for downstream analyses. The abundance of transcripts was calculated using the RSEM (RNA-Seq by Expectation–Maximization) approach [23], which estimates the abundance of genes and isoforms (expression levels) based on the mapping of RNAseq reads to the Trinity assembly. Estimates for read counts (reads originating from a particular gene), which are equivalent to a gene’s expression level, were provided using the expectation-maximization method. For abundance estimates, RSEM 1.2.7 was employed.

### 2.5. Identification of Differentially Expressed Genes (DEGs)

The Trimmed Mean of M-values (TMM) was employed as a normalization approach [24], and the differential expression tool edgeR v. 3.11a Bioconductor package was applied to identify pair-wise DEGs [25]. The counts per gene were normalized to Count per Million (CPM) [24]. The CPM-normalized data were then transformed with log2 using an offset of 1. Genes with at least 1 CPM were selected.

The transcripts represented by a *p*-value of ≤0.01, False Discovery Rate (FDR) of <0.01, and Log2Fold changes (Log2FC) ≥2.0 for up- and ≤−2.0 for downregulated DEGs were declared as significant in the four pair-wise comparisons across tissues and tested cvs (Supplementary Table S3). Among the tissue-specific DEGs, root and FPL tissue were separately compared between tested cvs and declared as upregulated in the western cv root or western cv FPL with a positive Log2FC, or otherwise upregulated in the eastern cv root and eastern cv FPL with a negative Log2FC. Similarly, cultivar-specific DEGs such as the western cv and eastern cv were compared between tissues of the developmental phases and declared as upregulated in ‘FPL’ with a positive Log2FC or otherwise upregulated in ‘root’ tissue for a negative Log2FC (Supplementary Table S3).

### 2.6. Functional Annotation of DEGs and Gene Ontology (GO)

Using a pair-wise comparison, DEGs were identified in the following tissues: root tissue (western cv vs. eastern cv) and FPL tissue (western vs. eastern cv); and cultivar-specific DEGs were identified in the following developmental stages: the eastern cv (FPL vs. root) and western cv. (FPL vs. root). These four pair-wise comparisons yielded a total of 17,566 DEGs expressed in any one of the four comparisons (Supplementary Tables S4–S7). These DEGs were subjected to functional annotation using Blast2Go (v 6.03) considering the taxonomy of flowering plants; the minimum BLAST expectation (E) value was 10^−5^, and the number of hits was 10. By connecting to the NCBI server, a Blastx-fast, InterPro scan was carried out to annotate each of the genes’ functional properties. Following the completion of functional annotation, GO categorization was carried out, along with KEGG pathway analysis, to classify DEGs into biological processes (BPs), cellular components (CCs), and molecular functions (MFs). Fisher’s exact test was performed with the total DEGs as a reference set (17,566) and the corresponding pair-wise DEGs as test sets to identify the enriched terms among the tissues and cultivars. The plant transcriptional factors database (PlantTFdb) was used to identify tissue- and cultivar-wise TFs.

### 2.7. qPCR Validation of Flowering Genes Across Cultivars

A total of twelve DEGs were subjected to qPCR validation across eastern and western cvs. The most stably expressed reference gene ‘ubiquitin’ was used as an endogenous control for qPCR analysis (Supplementary Table S8). Two biological and two technical replicates were used for relative expression quantification employing a 2^−ΔΔCt^ (Log2FC) method using a reference gene as an internal control. The Log2FC between the eastern and western cv of qPCR data and RNAseq data was compared for the validation of the gene expression results using a linear regression method (Supplementary Figure S6).

## 3. Results and Discussion

### 3.1. Phenotypic Changes During Reproductive Transition

The root morphological differences between the tested cvs are detailed in [10]. β carotene content shows significant differences between the cultivars and the western carrot has a higher carotene content than the eastern cvs [10]. For the flowering transition parameters of petiole thickness (mm), the length of the petiole (cm), days to flowering, and bud thickness (mm), significant differences were observed between cvs and between a cold room and field conditions (Table 1). The western cultivar, being biennial, did not show a floral transition in natural field conditions. However, its flowering transition ability was higher in a cold room under long-day conditions with far-red light exposure for 16 h a day, whereas, the eastern cv, having an annual habit, showed an earlier flowering transition in field conditions than in a cold room. The number of days to induce flowering in the western type in the current study was much earlier than in the previous research [11] due to the effect of light quality. Hence, the reproductive switch in carrots is highly influenced by the genotype, number of days of cold treatment, day length, and light quality [11].

### 3.2. De Novo Transcriptome Assembly

Modern western carrots derived from eastern cvs probably reduced their genetic diversity due to a domestication bottleneck and selection pressure, as noted by Iorizzo et al. [6]. However, there is greater genetic diversity and genetic differentiation between these two cvs, as mentioned in some reports [6,13,26].

The de novo assembly comprised 141,334,318 bases, 116,215 transcripts, and 49,521 unigenes from 12 RNAseq libraries (Supplementary Table S1 and Table 2). The N50 contig length was 1843 nt, with an average and median contig length of 1216.50 nt and 854 nt, respectively. The final assembly after redundancy removal through CD-HIT-EST retained 86,256,893 bases, 75,399 transcripts, and 48,394 genes (unigenes) with an N50 contig length of 1827 nt and a GC content of 39.32% (Table 2). To analyze the quality of the de novo assembly, we aligned 12 libraries back to the generated assembly and found that over 90% of the reads were successfully aligned (Supplementary Table S2). The BUSCO completeness assessment showed a completeness rate of over 90% (Supplementary Figure S1), indicating superior assembly quality for downstream analysis. The contig length of assembled transcripts ranged from 187 nt to 19,377 nt, and the most abundant sequences were in the range of <500 nt and 1000–2000 nt (Supplementary Figure S2). The results demonstrated that the assembly was of good quality for DEG analysis. The amount of data used in the study was higher than in some previous carrot transcriptomics [27,28,29,30]. The generated assembly was appropriate for identifying differentially expressed genes and isoforms across tested tissues and cultivars.

### 3.3. Identification of DEGs and Functional Annotation

**Figure 2:**
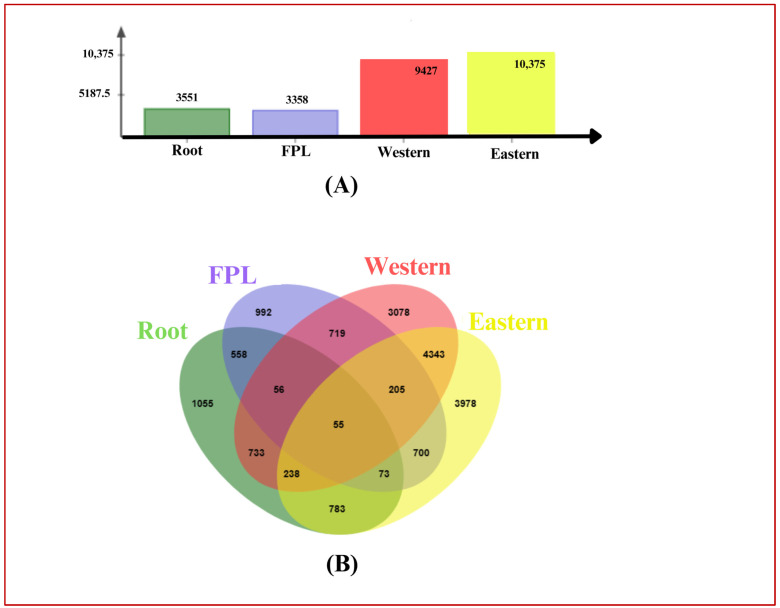
Pair-wise, unique, and common DEGs. (**A**) Number of DEGs identified for ‘root’ (eastern vs. western root) ‘FPL’ (eastern vs. western FPL), ‘western cv’ (root vs. FPL in western cv), and ‘eastern cv’ (root vs. FPL in eastern cv), represented in bar graphs. (**B**) The number of unique and common DEGs identified across tissues and cultivars.

There was a greater number of significant DEGs in cultivar-specific comparisons (over 9000) than in tissue-specific comparisons (less than 4000), as shown in Supplementary Table S3 and Figure 1 and Figure 2. The numbers of DEGs in the ‘Root’ and ‘FPL’ tissues were 3551 and 3358, respectively (Supplementary Tables S4 and S5, Figure 1A,B and Figure 2A). Meanwhile, the numbers of DEGs in the ‘western’ and ‘eastern’ cultivars were 9427 and 10,375, respectively (Tables S6 and S7, Figure 1C,D and Figure 2A). In root tissue, 1712 DEGs were upregulated in the western cv, and 1839 were upregulated in the eastern cv (Figure 1A). In FPL tissue, 1837 were upregulated in the western cv and 1521 were upregulated in the eastern cv (Figure 1B). Regarding the cultivar-specific DEGs, the western cv had 6420 and 3007 upregulated in FPL and root tissues, respectively (Figure 1C). On the other hand, the eastern cv had 6938 and 3437 upregulated in FPL and root tissues, respectively (Figure 1D).

**Figure 3:**
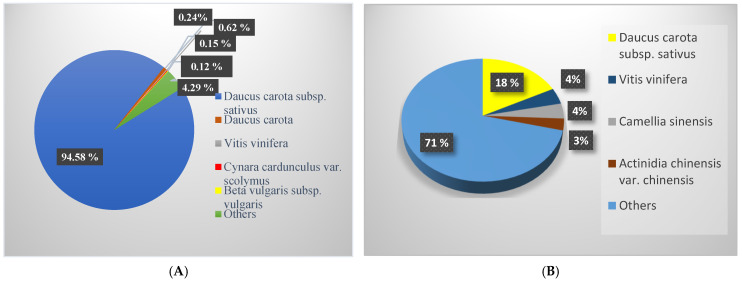
Details of BLAST hits of 17,566 DEGs with *p*-value 0.01 and Log2FC |±2|; (**A**) top five species BLAST hits; (**B**) species distribution of the BLAST hits.

Considering the presence of DEGs in at least one pair-wise comparison, we identified a total of 17,566 DEGs with over 85% functional annotations in the Blastx and InterPro databases (Supplementary Table S3). Out of those, 55 DEGs were identified in all four pair-wise comparisons, 1055 were unique to root tissue, 992 were specific to the FPL, and 558 were present in both tissues (Figure 2B). Additionally, 3078 DEGs were found to be specific to western cvs, 3958 were specific to eastern cvs, and 4343 were common to both (Figure 2B). The top species hit for DEGs was *D. carota* subsp *sativus* (94.58%), followed by *D. carota* and *Vitis vinifera*, among others (Figure 3A). The majority of DEGs were annotated for *D. carota* subsp *sativus*, followed by *V. vinifera*, *Camelia sinensis*, and other species (Figure 3B).

### 3.4. Identification of Transcription Factors

Over 85% (14,784) of the DEGs were annotated and identified with a putative function out of a total of 17,566 DEGs (Tables S4–S7). The significant differences between examined cvs or tissues included genes involved in carotenoids and flowering-related genes, photosynthesis, stress response, phytohormones, or metabolism-related pathways (Supplementary Tables S4–S7). The functional annotation of DEGs and GO categorization across tissues demonstrated a higher accumulation of ‘photosynthetic cascades’ in western cv root tissues and more of ‘auxin response’, ‘defense-response’, ‘disease-resistance’, and ‘cell wall biosynthetic genes’ in eastern cv root tissue. The photosynthetic response genes were co-expressed with carotenoid biosynthetic pathway genes in the previous transcriptome research involving western cv root tissue [6,10,30,31]. However, this is the first report on a comparison with the eastern cv across two developmental stages. In FPL tissue, the abundant expression of terpene synthase, protein kinases, ABC transporters, MADS-box proteins, and zinc finger proteins may be important in signaling, floral transition, and reproductive organ development, which were upregulated in both cvs.

**Figure 4:**
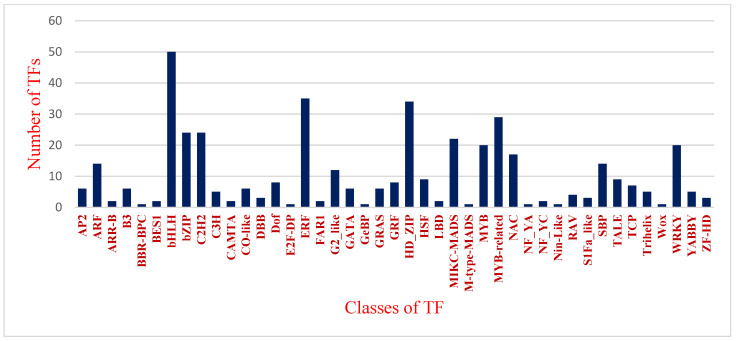
Classes of transcriptional factors differentially expressed across eastern and western cvs.

The PlantTFdb identified a total of 433 transcripts belonging to 43 classes as transcriptional factors from total DEGs (Figure 4). TFs such as bHLH (50), ERF (35), HD-Zip (34), and SBP, five each from bZIP- and MYB-related (29) classes, were abundant. Other TF classes, viz., bZIP (24), C2H2 (24), MIKC-MADS (22), MYB (20), and WRKY (20), were found more frequently. TFs involved in the flowering pathway (MIKS- and M-type-MADS, Wox, WRKY, Dof, CO-like) and plant stress response to a/biotic stresses (WRKY, bHLH, NAC, bZIP, MYB, AP2/ERF) were identified with variable expression in tested cvs, indicating their diversity among the tested cvs. WRKY, MYB, ERF, and NAC are the major TF families known to mediate stress mechanisms by regulating secondary metabolites [31,32]. TF families such as bHLH, bZIP, and MYB are involved in growth and development in response to the environment [32]. TFs such as DBB and GRF are known to play a key role in photosynthesis, cell division in the cambial meristem, and lateral organ development, whereas HSF acts as an osmo-protectant in regulating soluble sugars and proline during cold stress [33,34]. The YABBY TF is known to play a vital role in the development of leaves and floral organs [34], while the B3 TF present in xylem tissue plays an important role in wood formation [10,35].

### 3.5. Gene Ontology (GO) Classification and KEGG Pathway Analysis

A total of 17,566 DEGs were classified into different categories, namely biological processes—BPs (67); cellular components—CCs (13); and molecular function—MF (28), as well as KEGG (95) pathways (Figures S3 and S4). Within the biological process (BP) category, the most common related terms were ‘cellular process’ and ‘metabolic process’, in addition to ‘biosynthetic process’, ‘response to stimulus’, ‘response to stress’, ‘regulation of various molecules’, ‘gene expression’, ‘regulation of expression’, ‘localization’, ‘transport’, and ‘protein modification process’. These terms indicate the role of DEGs in various physiological processes, such as growth, development, and the transition from the vegetative to the reproductive stage (Supplementary Figure S3). The cellular component (CC) terms also highlight the involvement of DEGs in the ‘cellular anatomical entity’, along with other components such as the ‘intracellular anatomical structure’, ‘membrane’, and ‘organelle’. Among the molecular function (MF) terms, ‘binding’, ‘catalytic activity’, ‘transferase activity’, ‘hydrolase activity’, and ‘kinase activity’ were the most common terms (Supplementary Figure S3).

The majority of DEGs in the KEGG pathways were related to the metabolic pathway. This study identified several key pathways, including the biosynthesis of secondary metabolites, carbon metabolism, plant hormone signal transduction, the biosynthesis of amino acids, ribosomes, plant–pathogen interactions, photosynthesis, and phenylpropanoid biosynthesis (Supplementary Figure S4).

KEGG pathways in the tissue-specific enrichment analysis identified several enriched pathways, such as ‘photosynthesis’, ‘carbon metabolism’, ‘Porphyrin and chlorophyll metabolism’, and ‘glycolysis’ in root tissue (Supplementary Figure S5A–D). Root tissue-specific pathways comprised ‘MAPK signaling’, ‘Glycolysis’, ‘pentose phosphate pathway’, ‘biosynthesis of amino acids’, and ‘plant hormone signal transduction’ (Supplementary Figure S5A), while FPL tissue was enriched with pathways including ‘protein export’, ‘photosynthesis’, and ‘nitrogen and glycerolipid metabolism’ (Supplementary Figure S5B). FPL tissue-specific KEGG pathways, such as ‘protein export’, the ‘flavonoid pathway’, the ‘phenylpropanoid pathway’, and ‘ABC transporters’, were also identified. Notably, the pathways of ‘photosynthesis’, ‘carbon metabolism’, ‘fatty acid degradation’, and ‘plant-pathogen interaction’ were common between both storage root and FPL tissues (Supplementary Figure S5A,B). Highlighting the source-to-sink transfer of photosynthates during flowering, a significant amount of carbohydrates is stored in its expanded taproots for the carrot plant to flower in its second year [3].

In both the tissues, ‘metabolic pathways’ and the ‘biosynthesis of secondary metabolites’ pathways were identified as the top enriched pathways. Additionally, ‘carbon fixation in photosynthetic organs’ and ‘carbon metabolism’ in the western cv were also among the top enriched pathways. In the eastern cv, the ‘photosynthesis pathway’ was also enriched (Supplementary Figure S5C). KEGG enrichment analysis revealed that the western cv had specific pathways like the ‘Phosphatidylinositol signaling system’ and ‘phenylpropanoid biosynthesis’ which were absent in the eastern cv (Supplementary Figure S5C). However, in the eastern cv, specific pathways such as ‘Fructose and mannose metabolism’ and ‘Ascorbate and aldarate metabolism’ were present (Supplementary Figure S5D). Interestingly, both tested cvs displayed enriched pathways such as ‘photosynthesis’, ‘carbon fixation in photosynthetic organisms’, ‘carbon metabolism’, ‘glycolysis’, the ‘pentose phosphate pathway’, the ‘biosynthesis of amino acids’, ‘fatty acid degradation’, ‘plant hormone signal transduction’, and the ‘biosynthesis of secondary metabolites’ (Supplementary Figure S5C,D).

### 3.6. Carotenoid Pathway Gene Variations: Directing the Synthesis of Carotene or Stress Hormones

The domestication of modern dark orange western carrots is considered one of the greatest milestones in the carrot crop improvement program. Carotenoid biosynthesis not only accumulates α- and β-carotene or lycopene but also plays a key role in photosynthesis, plant development, and photorespiration and the diversion to the ABA and apocarotenoid pathways [16,36]. Hence, the discussion of carotenoid biosynthesis goes beyond its relevance in human nutrition, encompassing its pivotal role in signaling and the reproductive switch for the development of climate-resilient, high-yielding carrot varieties rich in carotenoids [36,37]. In high-carotenoid cvs, α and β carotenoids are accumulated in high quantities [37], and they are also co-expressed with photosynthetic genes [6,10].

**Figure 5:**
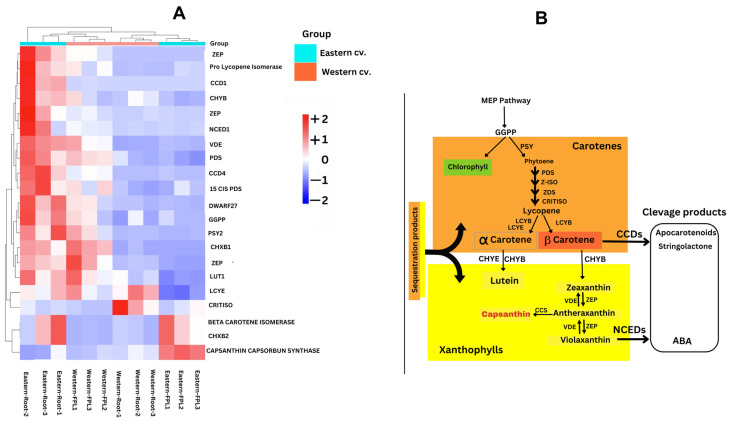
(**A**) Heat map of key carotenoid pathway genes differentially expressed across eastern and western cv root and FPL tissues based on TPM values. (**B**) Putative carotenoid pathway highlighted with identified genes.

In the present study, key enzymes such as phytoene synthase (PSY), carotenoid isomerase (CRTISO), and lycopene epsilon cyclase (LCYE) and zeaxanthin epoxidase (ZEP) were found to have a higher expression in western cv root tissue (Figure 5A,B), while β-carotene hydroxylase (CHYB) and violaxanthin de-epoxidase (VDE) were upregulated in eastern cv root tissue, probably resulting in a higher accumulation of these nutritionally important α- and β-carotenes in the western cv [6,26]. Another enzyme, β-carotene hydroxylase (CHXB1) or β-carotene 3 hydroxylase (CHXB1), adds oxygen to the cyclic β-ring of cyclic carotenes to produce the yellow pigment Xanthophyll [16]. CHXB1 was higher in eastern root and FPL tissues, revealing its probable role in the yellow xylem of the low-carotenoid eastern cv. Carotene epsilon-monooxygenase (LUT1), which catalyzes the reaction from α-carotene to α-cryptoxanthin [38], known as an immediate precursor of lutein, was found in the eastern cv and could explain an accumulation of yellow pigment in the xylem tissue of its root tissue. Carotenoid 9,10(9′,10′)-cleavage dioxygenase 1 (CCD1) is involved in the production of apocarotenoids, important compounds that regulate the carotenoid turnover, deciding the color and aroma, and is involved in the production of two phytohormones, ABA and stringolactones, both involved in abiotic stress responses [16]. Therefore, it is a rate-limiting enzyme for the conversion of β-carotene to enter apocarotenoid synthesis. A higher accumulation of CCD1 in the FPL of the eastern cv and CCD4 in the FPL of the western cv suggests their role in the regulation of flower transition by producing the stress hormone stringolactone (Figure 5A,B). The overexpression of CCD4 in orange carrots results in a pale yellow color and decreased content of α and β carotenoids due to the production of β-ionone, a type of apocarotenoid [8,38]. NCED1 is an important enzyme that cleaves 9-cis violaxanthin to produce xanthoxin, which further modifies ABA, known as an important hormone in mediating the abiotic stress response through the ABA pathway [8,16]. The expression of NCED1 was higher in the FPL tissue of the eastern cv than in the western cv, indicating the successful production of ABA and its probable role in triggering flowering through endogenous ABA synthesis in the eastern cv while stabilizing the plant response under stress [39]. The expression of another important enzyme, namely β-carotene isomerase and β-carotene isomerase D27, was restricted to FPL tissue in both tested cvs. An extensive study on cis/cis-β-carotene isomerase, D27 [40], elaborated its role in the isomerization of trans into 9-cis β-carotene, initiating the stringolactone pathway and affecting the ABA pathway. Hence, the presence of CCD and NCED would facilitate plants in triggering the ABA pathway as a defense mechanism under abiotic stresses, especially during the early stages of floral transition and floral primordia production. Notably, after the formation of the β-carotene compound, the downstream enzymes of the carotenoid pathway that links to the ABA and stringolactone stress-responsive pathways, such as CCD, NCED, VDE, ZEP, and CHYB, were more active in FPL tissue than in root tissue (Figure 5A). This difference was higher in the eastern cv than in the western cv (Figure 5A). The carotenoid biosynthetic process, confirmed by the presence of DEGs in the present work, is represented in Figure 5B.

In the present study, we found 19 key carotenoid pathway genes involved in carotenoid biosynthesis, revealing their essential role in the carrot life cycle. Well-developed carrot storage roots will have higher levels of α- and β-carotene, which would be utilized in the signal transduction pathway for the synthesis of phytohormones such as ABA and strigolactone during the floral induction stage (Figure 5B). Our study presumes that a higher accumulation of these later signaling genes in the eastern cv probably leads to the withstanding of various abiotic stresses and an easier floral transition.

### 3.7. Expression or Regulation of Flowering Repressors in Deciding the Floral Transition Time in Carrots

**Figure 6:**
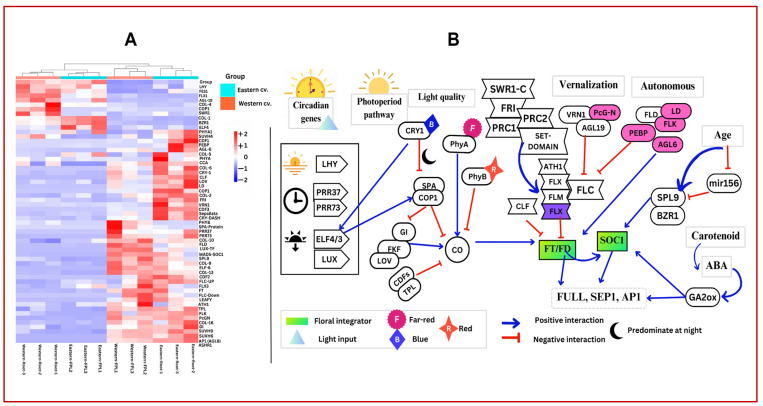
(**A**) Heat map of key flowering genes identified in the present study based on TPM values. (**B**) Putative illustration of flowering pathway network proposed for eastern cv based on identified DEGs. Highlighted in the pink are genes that predominated especially in the eastern cv; genes highlighted in the green box are key flowering integrator genes critical for reproductive transition.

A biennial habit of flowering and vernalization are two recent domestication traits in western dark orange carrots [10,41]. Carrots have been classified into long-day and day-neutral crops [11]. A vernalization of 0 to 10 °C, coupled with long-day conditions, successfully induces flowering in biennial carrot cvs [42]. Wild and eastern carrot cvs have variable photoperiod requirements; however, there is no significant information about the photoperiod requirement for flowering transition. In the present study, flowering was induced by growing both eastern and western cvs in a phytotron facility maintained at 4 °C to 8 °C in the presence of 16 h of far-red light and 8 h of darkness. A higher number of DEGs involved in the flowering pathway with variable abundance and expression patterns were identified in tested cvs (Figure 6A). Therefore, this study marks the foundational presentation of putative flowering pathway networks independently operative in eastern and western carrots. These findings shed light on the roles of these genes in the flowering process, drawing from extensive research on flowering pathway regulation in *Arabidopsis* and other plants. The coordinated network of the circadian clock and photoperiod pathway through CONSTANS (CO)-mediated floral induction and FLC repression mediated by vernalization and autonomous pathways is elaborated here in two globally important carrot cvs (Figure 6B).

Circadian clock genes have evolved systematically to coordinate the photoperiod and light quality genes for the regulation of CONSTANS, a precursor that directly activates the floral integrator FT [43]. A circadian clock gene, namely the morning gene, light-dependent short hypocotyl (LHY), was found to be upregulated in the western cv root. Remaining clock genes, viz., mid genes, Pseudo Response Regulator genes (PRR37, PRR73), evening genes, early flowering genes (ELF4, ELF6), and the night gene LUXARRHYTHMO (LUX) were found to be upregulated in FPL tissue in the western cv (Figure 6A), while in the eastern cv, LHY, ELF3, ELF4, PRR37, and PRR73 were present in FPL tissue and ELF4 in root tissue. These clock genes act as oscillators and play a major role in the regulation of large sets of genes for metabolic and physiological processes [43]. As a substrate adaptor, EARLY FLOWERING 4 (ELF4) enables contact between CONSTITUTIVELY PHOTOMORPHOGENIC (COP1) and GIGANTIA (GI) in the photoperiod pathway (Figure 6B). COP1, which encodes for E3 ubiquitin ligase, coupled with the suppressor of phyA-105 (SPA), degrades CONSTANS (CO) during darkness and was also upregulated in the FPL tissue of both the cvs (Figure 6A). ELF4/ELF6 helps in connecting the circadian rhythm genes with photoperiod-responsive genes by activating COP1 and SPA. This COP1/SPA complex, a photomorphogenesis-repressing complex that inactivates GI during the dark, is important in degrading CO, a central photoperiod flowering regulator [44]. However, in the presence of photoreceptors like PHYA or CRYPTOCHROMES (CRY), this COP1/SPA complex will be degraded under light to activate CO via the activation of GI [44] and CRY1; a blue light receptor will probably degrade this complex as we found a higher expression of CRY1 and GI in the tested cvs (Figure 6A,B). Another photoreceptor, PHYTOCHROME B (PHYB), a thermo-tolerant red light receptor, also upregulated in the eastern cv, could have a role in regulating the de-etiolation response in addition to the repression of COP1 [45]. The photoactive LOV domain present in the F-box Kelch repeat protein (FKF) acts as a photoperiodic blue light receptor [45]. LOV domains and the FKF were found to be more abundant in eastern cv FPL than root tissue (Figure 6A). The expression of GI will be stabilized by the binding of the FKF to form a GI/FKF/LOV complex under light [44,45]. The F-box Kelch repeat protein (FKF) with its blue light receptor LOV domain attached to GI causes it to be released from the repression complex, then promotes the transcription of CO (Figure 6B). In addition, cryptochrome-1 (CRY1—a blue light receptor) and cryptochrome DASH are upregulated in FPL tissue to stabilize the GI/FKF/LOV complex in both the cvs (Figure 6A,B). In addition to CRY, another photoreceptor, PHYTOCHROME A (PHYA), a far-red light receptor, was found in the eastern cv. Hence, there has been an abundant expression of CONSTANS (24 transcripts in western and 27 transcripts in eastern cv FPL tissues). CO homologs, viz., CO-1, CO-2, CO-4, CO-5, CO-6, CO-9, CO-10, CO-13, and CO-16, were abundant in both the cvs despite the presence of repressor-like cyclin DOF factors (CDF 1, 2, and 3), PHYB (eastern cv) and TOPLESS (TPL, TPL-1, TPL-4) [46,47], leading to successful FT expression and floral transition (Figure 6A,B). Interestingly, we found four more novel CO homologs (CO-10, -16, -6, and -9) from this study than in previous reports on carrots [48]. This network would have probably induced the floral integrator gene, flowering locus T (FT), expression in FPL tissue, as shown by its upregulation in FPL tissue in both the cvs. Interestingly, we found another floral integrator gene, i.e., flowering locus D (FD), in addition to FT and FT-interacting proteins (syntaxin and Quirky) in the root tissue of the eastern cv. It is reported that FT found in phloem companion cells in the root is mediated by an FT interaction with syntaxin and Quirky [49,50]. FD interacts with endogenous signaling complexes including sugar and hormones to induce flowering [51] and was expressed specifically in the eastern cv. Important floral integrator genes downstream to FT, i.e., SUPPRESSION OF OVEREXPRESSION OF CONSTANS (SOC1), were highly abundant in the FPL tissue of both the cvs (Figure 6A). Hence, there was successful production of the meristematic identity genes SEPALLATA, LEAFY/Floricaula, AGAMOUS-like MADS-box protein 8 or AGL8 (also known as AP1 or FRUITFULL), and APETALLA-2 (AP-2) and AP3 in the FPL tissue of both the cvs. (Figure 6A,B).

Both floral repressors and promoters via vernalization have been extensively studied in *Arabidopsis* and a few other crops [50], and we have captured both of them in the current study. For instance, FRI is a key regulator of FLC and a repressor of flowering that experiences epigenetic changes during vernalization [48,50]. In addition, FRI promotes the expression of FLOWERING LOCUS C (FLC) and represses flowering before cold treatment by chromatin modifiers like histone ubiquitination and histone lysine methylation. Histone lysine N-methyltransferase (Mtase) ASHR1 (in root tissue) and histone lysine N-methyltransferase H3 lysine-9-specific SUVH4-like methyltransferase (in the root and FPL tissue of the western cv), known as SET domain proteins encoding methyltransferases (MTases), were upregulated in the root and FPL tissue of the western cv (Figure 6A). These SET domain proteins are known to be potentially involved in H3K4 and H3K36 methylations [52]. Similarly, FRI activation by SET domains encoding MTases such as SUVH6, SUVH9, and ASHR1 were present in the eastern cv (Figure 6A). Under cold and lower temperature exposure during the winter, FRI undergoes a series of epigenetic changes (e.g., histone and chromatin modifications by the polycomb repressive complex (PcG N-terminal) including VRN genes), repressing FLC and promoting flowering [53]. FLC is a MADS-box transcription factor that functions with its upstream activator FRIGIDA (FRI) to prevent flowering during the development of the edible storage roots of both the cvs (Figure 6A). FRI produces FLC, which regulates the timing of flowering. We found upregulation of the transcripts encoding FLC and FRIGIDA in FPL tissue. In the western cv, we found a higher expression of FRI in FPL tissue in addition to the SWR1-C gene (Figure 6 B), a positive regulator of FLC [42], which protects plants from reproductive transition during the vegetative phase. FLC repressor genes, viz., VRN1, VIN3, VRN2, AGL19, PcG N-terminal, and PRC1 and 2, are known to become activated upon vernalization and degrade FLC expression [50,52]. We found a higher expression of VRN1 in both root and FPL tissues, indicating the essentiality of vernalization in repressing FLC for successful floral induction in the western cv [11]. Prolonged vernalization activated VRN1 with polycomb repressors (PcG N-terminal) and AGL 19 in the eastern FPL tissue (Figure 6A). While VRN-1 and AGL-19 were found to be abundant in the western cv (Figure 6A,B). FLC repressors such as VRN1, a vernalization enhancer polycomb N-terminal (PcG N-terminal), and agamous-like MADS-box protein, AGL-19, were upregulated in the eastern cv and may form a complex to regulate FRI. Apart from FLC, we have found that FLC-like genes, flowering locus M, FLM (also known as AGL19), flowering locus X (FLX), agamous-like MADS-box AGL27, and homeobox protein (ATH1) in both the cvs. These could have probably been repressed during vernalization and successfully induced the expression of the flowering integrator gene FT (Figure 6A,B). Upon vernalization, FLC repressors such as VRN1, PcGN, and AGL 19 were upregulated to inactivate FLC and FLC-like proteins for successful floral induction. Apart from these vernalization, photoperiod, and circadian clock genes, we found one autonomous pathway gene, flowering locus D (FLD), in both the root and FPL tissues of the western cv that is known to repress FLC without the need for vernalization [53]. However, the overdominance of FRI would probably override FLD expression in the western cv [50], and would not promote flowering autonomously.

The eastern cv may induce flowering without vernalization as this trait is a result of secondary domestication changes only in the western cv [9]. Hence, probably, the eastern cv has an adaptive mechanism/endogenous regulation by potential autonomous genes that override FRI and repress FLC independently. Several autonomous pathway genes were identified in the eastern cv, viz., FLD, flowering locus K (FLK), and Luminidependens (LD) in addition to agamous-like MADS-box AGL 6 [53], that regulate histone modification and transcription to regulate FLC (Figure 6A,B). Additionally, the presence of PHOSPHATIDYLETHANOLAMINE-BINDING PROTEIN (PEBP) in the eastern cv reveals its crucial role in orchestrating the vegetatively regulated transition to reproductive stages in geophytes like carrots [54]. PEBP is also an important conductor of reproductive development in geophytes in response to environmental factors (especially in the tropics) using endogenous cues [42].

As the plant ages, concentrations of the SQUAMOSA PROMOTER BINDING-LIKE (SPL) proteins increase. SPL proteins were found to be abundant in both eastern and western cvs (Figure 6A). SPL proteins are negatively regulated by mir156 during the vegetative phase, making plants insensitive to vernalization. Hence, upon vernalization with aging, SPL6, 7, 8, and 9 proteins were found to be abundant between cvs. BRASSINAZOLE-RESISTANT 1 (BZR1) interacts with SPL9, promoting vegetative phase transition (Figure 6A,B). The gibberellin, autonomous, and age pathways regulate flowering by responding to internal signals [47,51]. In addition, in the shoot apical meristematic region, the ABA-GA balance is maintained by ABI4 (abscisic acid insensitive-4). The transcriptional levels of ABI and ABA hydrolases were found to be upregulated more in the eastern than in the western cv (Supplementary Table S4 and Figure 6B). ABA–GA cross-talk in the shoot apical meristematic region can induce Gibberellin-20oxidase (GA-20ox) as a signal for vegetative phase change [39]. The abundant expression of the downstream floral integrator gene MADS-box SOC1 and the meristem identity genes SEPALLATA, AGL8 (encodes AP1 and FRUITFUL), LEAFY/Floricaula, and APETALLA3 in FPL tissues was essential for floral organ development immediately after flowering induction (Figure 6A,B). Therefore, we propose that both the CONSTANS-mediated expression of FT and the FLC-mediated repression of FT are supported by external and internal cues that are essential in orchestrating the successful transition from the vegetative to the reproductive phase in carrots. In the present study, we found a majority of the genes in the circadian, photoperiod, and vernalization pathways, indicating their probable role during the reproductive transition in carrots. Our results demonstrate that, in the eastern cv, apart from the photoperiod response pathway, the autonomous pathway, and the gibberellic acid-mediated pathway, sugar signaling is well-established endogenously to promote floral transition with a minimal need for external cues. Consequently, as an annual plant, the eastern carrot undergoes floral transition easily without vernalization.

### 3.8. Validation of DEGs in qRT-PCR

**Figure 7:**
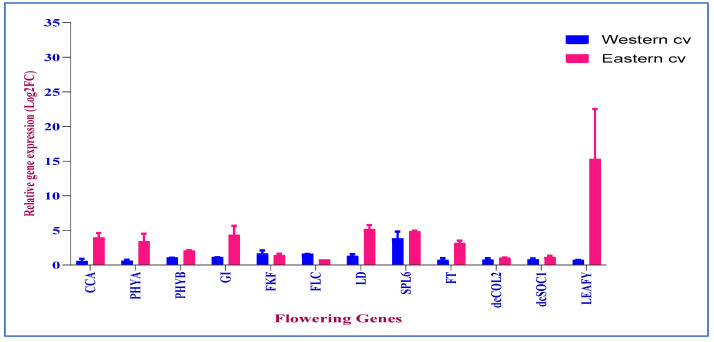
Relative expression changes in 12 key flowering genes across eastern and western carrots in Log2FC from qRT PCR.

Log2FCs obtained from RNAseq and qRT-PCR experiments across the eastern and western cultivars were compared to validate the significance of the identified DEGs (Supplementary Table S8). The results for twelve genes compared between the two experiments showed a concordant relationship between both the experiments with an R^2^ value of 0.64 (Supplementary Figure S6), indicating the reliability and precision of the generated RNAseq data and confirming the quality of de novo assembly (Supplementary Figure S6). A distinct gene expression pattern was found in eastern and western cvs for the tested flowering genes, indicating that distinct pathways operate in deciding the time of floral transition in both cultivars (Figure 7). The functional validation of key flowering genes such as CCA, PHYA, PHYB, GI, FKF, FLC, LD, SPL6, FT, dcCOL2, dcSOC1, and LEAFY representing all the flowering pathways across two cultivars indicates their critical role in deciding the flowering time in carrots (Figure 7).

### 3.9. Application of Findings in This Study

This study presents new genomic information characterizing the flowering pathway for carrots in comprehensive detail, based upon the evaluation of flowering in both annual eastern and biennial western carrots, and it provides previously unreported insights into the relationships between phytohormone-synthesizing genes in the carotenoid pathway for pigment accumulation. With this, insights into the molecular underpinnings of traits involved in the early stages of carrot domestication are more clearly understood. Additionally, the relationships between the molecular biology of floral initiation, abiotic stress, and carotenoid pigment accumulation are now understood to be more closely related to each other than was previously appreciated. Since flowering, stress tolerance, and nutritional quality are primary traits addressed for carrot improvement today, these findings are relevant to the agricultural research community, including not only carrots, but also research underway in other crops, and perhaps especially root crops.

## 4. Conclusions

Through a de novo assembly and analysis of differentially expressed genes, valuable information has been generated to understand the genetic/molecular changes that probably occurred during secondary domestication from eastern to western carrot cvs. The chosen plant materials, tissues, and methodology were particularly relevant in explaining the evolutionary changes and selection patterns. Western carrot cvs are known for their efficient photosynthesis, leading to higher carotene levels in the storage root. However, it has probably undergone selection pressure against endogenous cues for the reproductive transition without external stimuli like vernalization. On the other hand, the eastern cv has highly active phytohormone-synthesizing genes in the carotenoid pathway and defense response genes with fewer photosynthetic genes during root production. The eastern cv relies on internal cues, such as the autonomous pathway, and GA-mediated floral induction without specific vernalization or photoperiod requirements. Comprehending these distinct pathways of carotenoid biosynthesis and flowering regulation is paramount for enhancing carotene levels in eastern carrots. A detailed understanding of the potential roles of CCD and/or NCED genes or autonomous pathway genes like FLK, FLD, LD, and PABP in both cultivars is essential for employing them in carrot breeding. The western cv is biennial and vernalization is obligatory to repress FLC to successfully switch to flowering. Hence, in biennial carrots, understanding the CONSTANS-independent and FLC-mediated repression of flowering is crucial to developing an alternative strategy to vernalization for the induction of flowering in tropical conditions.

## Figures and Tables

**Table 1:** Phenotypic changes during the flowering transition of eastern and western cultivars in cold room and field conditions.

Parameters	Cold Room (Long Day)		Field Conditions (Short Day)	
	Eastern Cv	Western	Eastern	Western
Plant height (cm)	48.33 ± 10.47 ^a^	44.67 ± 3.84 ^a^	43.67 ± 1.86 ^a^	36.00 ± 4.00 ^a^
No. of petioles	12.33 ± 0.66 ^a^	10 ± 1.15 ^a^	11.33 ± 0.88 ^a^	9.33 ± 0.33 ^a^
Length of petiole (cm)	23.00 ± 3.51 ^a^	21.67 ± 2.72 ^a^	34.18 ± 0.29 ^b^	28.56 ± 0.40 ^a^
Primary petiole thickness	2.75 ± 0.20 ^a^	3.65 ± 0.26 ^b^	4.81 ± 0.16 ^b^	4.42 ± 0.22 ^b^
Days to flowering, western	62.33 ± 0.66 ^a^	0.00 ^c^	22.33 ± 1.17 ^b^	0.00 ^c^
Bud thickness (mm)	3.44 ± 0.58 ^ab^	3.87 ± 0.42 ^a^	2.93 ± 0.06 ^a^	0.00 ^b^

Comparison within cold room and field conditions and comparison between conditions for specific genotypes was performed with paired *t*-test at *p* ≤ 0.05. Similar letter indicates non-significance and the different letters indicate a significant differences.

**Table 2:** Details of de novo assembly obtained from root and FPL tissues of eastern and western carrots from 12 RNAseq libraries.

Trinity Assembly Output	De Novo Assembly	
	Total Assembly Output	After CD HIT-EST
Total assembled bases	141,334,318	86,256,893
Total transcripts	116,215	75,399
Total ‘genes’	49,521	48,394
Percent GC	39.68	39.32
Contig N50 (nt)	1843	1827
Median contig length (nt)	854	746
Average contig (nt)	1216.15	1144.01

## Data Availability

The raw data as SRA files are available in the NCBI database as a Bio project with the project ID PRJNA913450.

## Supplementary Materials

The following supporting information can be downloaded at https://www.mdpi.com/article/10.3390/genes15111462/s1. Figure S1: BUSCO completeness assessment of denovo assembly against Eudicots lineage with 2326 BUSCOs. Figure S2: Details of the Contig length of De novo assembly. Figure S3: Gene ontology classification of 17,566 common DEGs identified across tissues and cultivars. Figure S4: KEGG Pathway analysis of total DEGs across tissues and cultivars. Figure S5: Top 20 KEGG pathways enriched in different tissues (A) Root tissue (B) FPL Tissue] and among cultivars (C) Western tissue (D) Eastern cv drawn in shinyGO0.77 using Daucus carota subsp sativus as a reference. Figure S6: Validation of DEGs identified in RNA seq by quantitative real-time PCR (qRT-PCR) based on Log2FC and linear regression method. Table S1: Summary of input reads, filtered and trimmed reads of 12 RNAseq libraries across two tissues (root and FPL) and two cultivars (Eastern and Western) in triplicates. Table S2: RNA seq read representation and Bowtie 2.0 alignment rate. Table S3: Pairwise comparison of DEGs and the details of functional annotation. Table S4: List of Up and downregualted DEGs of Root tissue across Western and Eastern carrot cultivars and their functional annotation, LogFC (>2 or <−2.0) and *p* values (0.01). Table S5: List of Up and downregualted DEGs of FPL tissue across Western and Eastern carrot cutlivars and their functional annotation, LogFC (>2 or <−2.0) and *p* values (0.01). Table S6: Annotation of DEGs of Western cultivar that are Up and downregualted across FPL and Root tissues @ LogFC (>2 or < −2.0) and *p* values (0.01). Table S7: Annotation of DEGs of Eastern cultivar that are Up and downregualted across FPL and Root tissues @ LogFC (>2 or <−2.0) and *p* values (0.01). Table S8: Details of Key flowering genes used for q-RTPCR validation.
