# Supplementary material for: Deciphering Carotenoid and Flowering Pathway Gene Variations in Eastern and Western Carrots (Daucus carota L.)
Source: Genes (Basel). 2024 Nov 13;15(11):1462. doi: 10.3390/genes15111462 (PMC11593857; doi:10.3390/genes15111462)
Supplement: Supplementary file 1 [file genes-15-01462-s001.zip › genes-3302407-Supplementary_Figures_Genes.pdf]

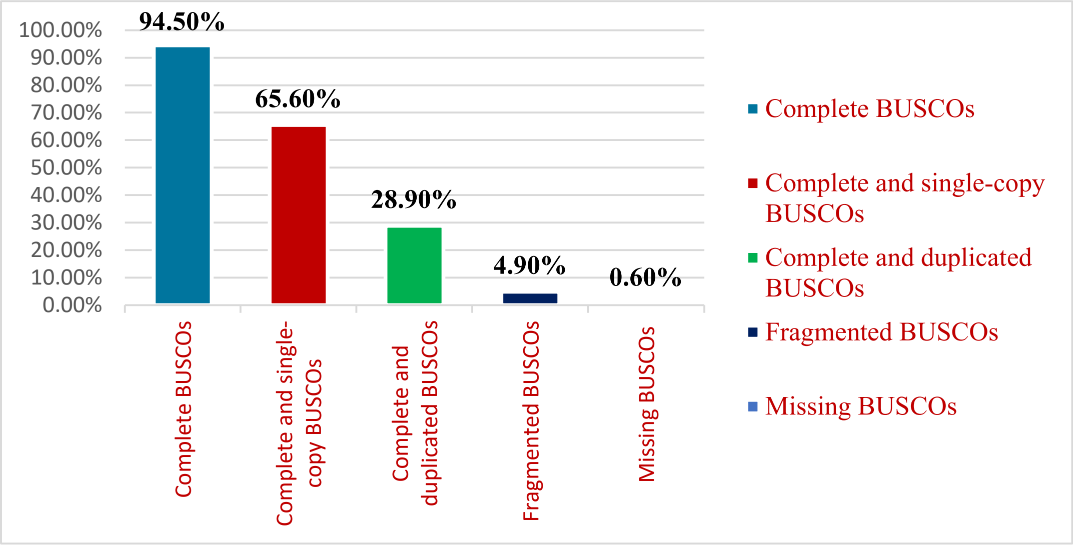

Supplementary Figure S1: BUSCO completeness assessment of denovo assembly against Eudicots lineage with 2326 BUSCOs

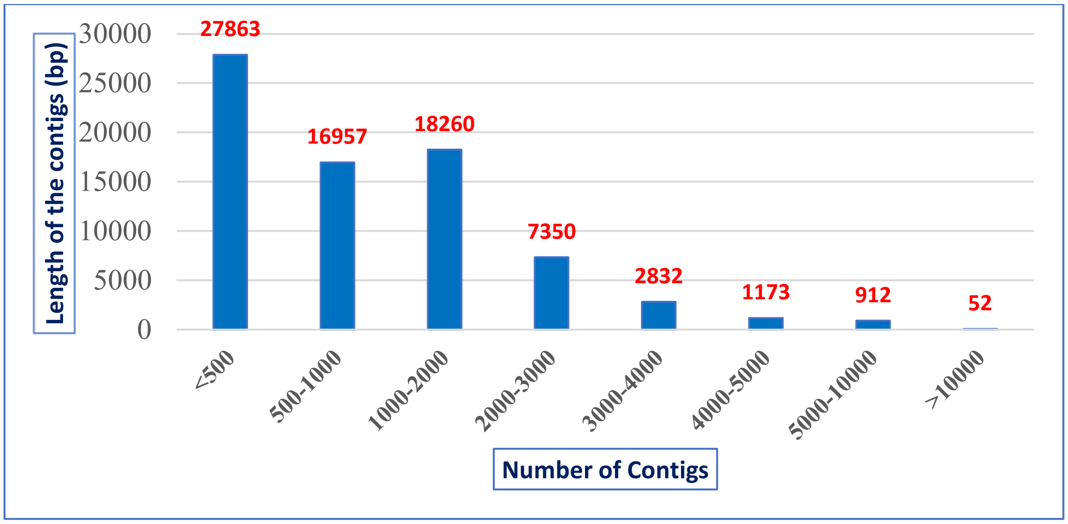

Supplementary Figure S2: Details of the Contig length of *De novo* assembly

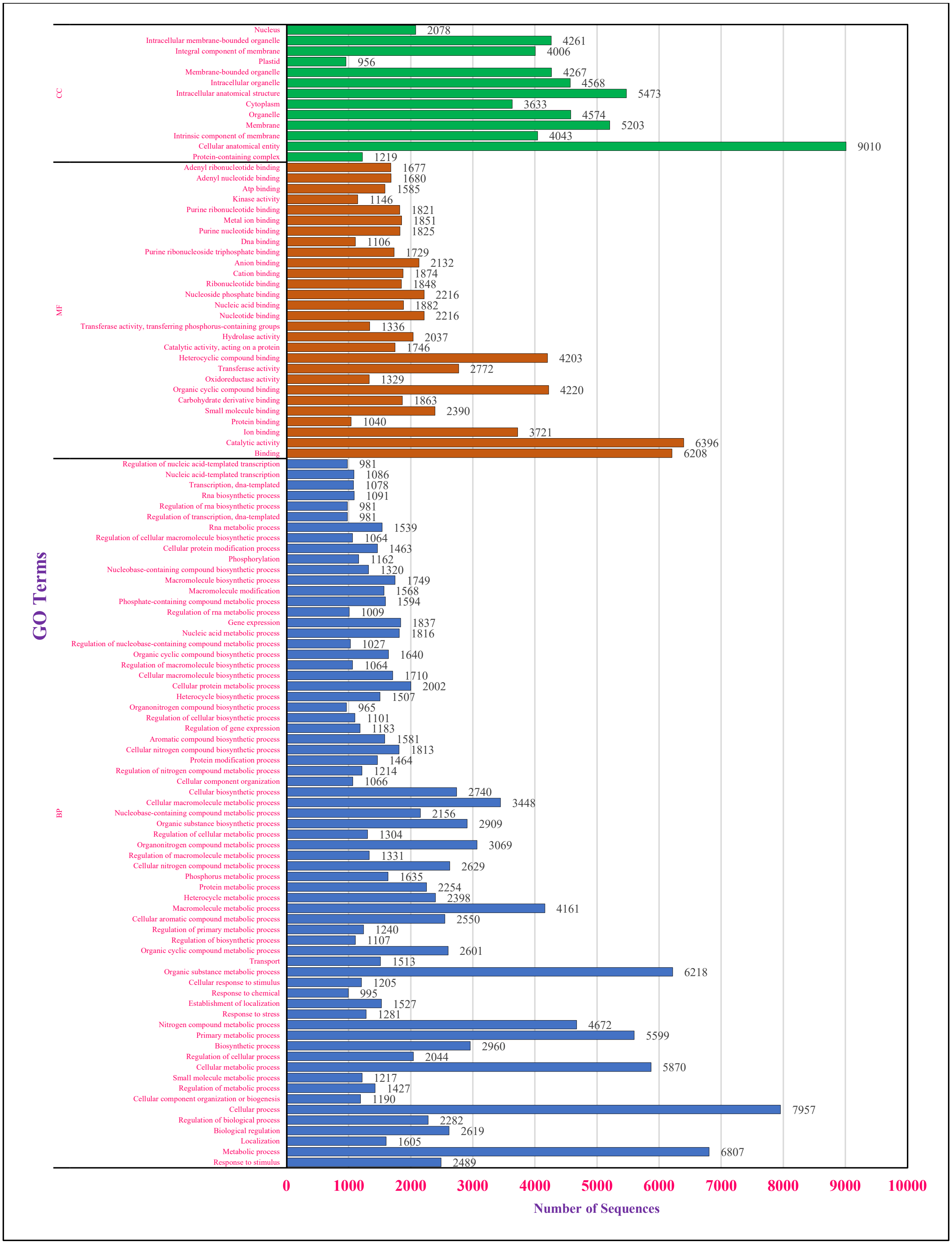

Supplementary Figure S3: Gene ontology classification of 17566 common DEGs identified across

tissues and cultivars

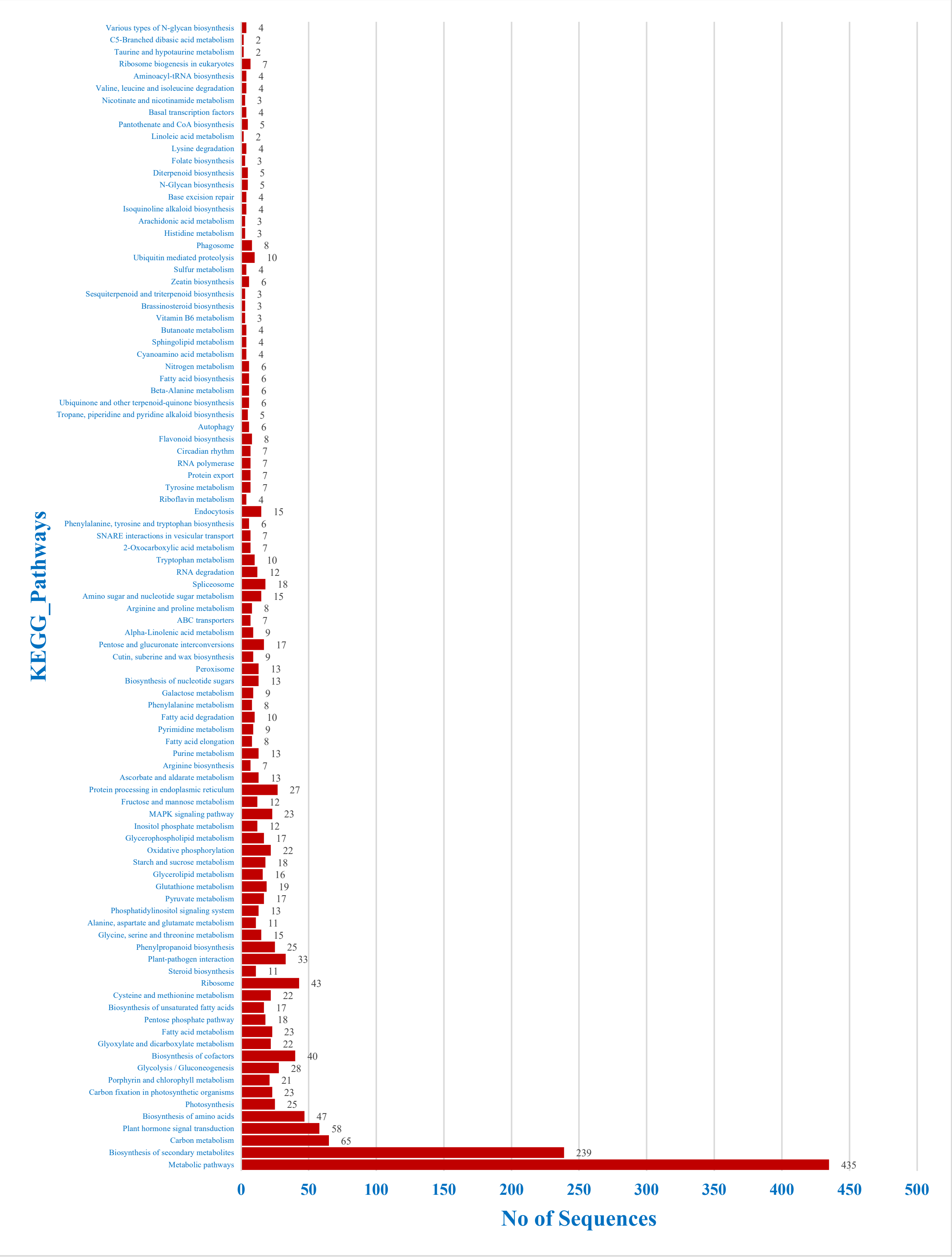

Supplementary Figure S4: KEGG Pathway analysis of total DEGs across tissues and cultivars

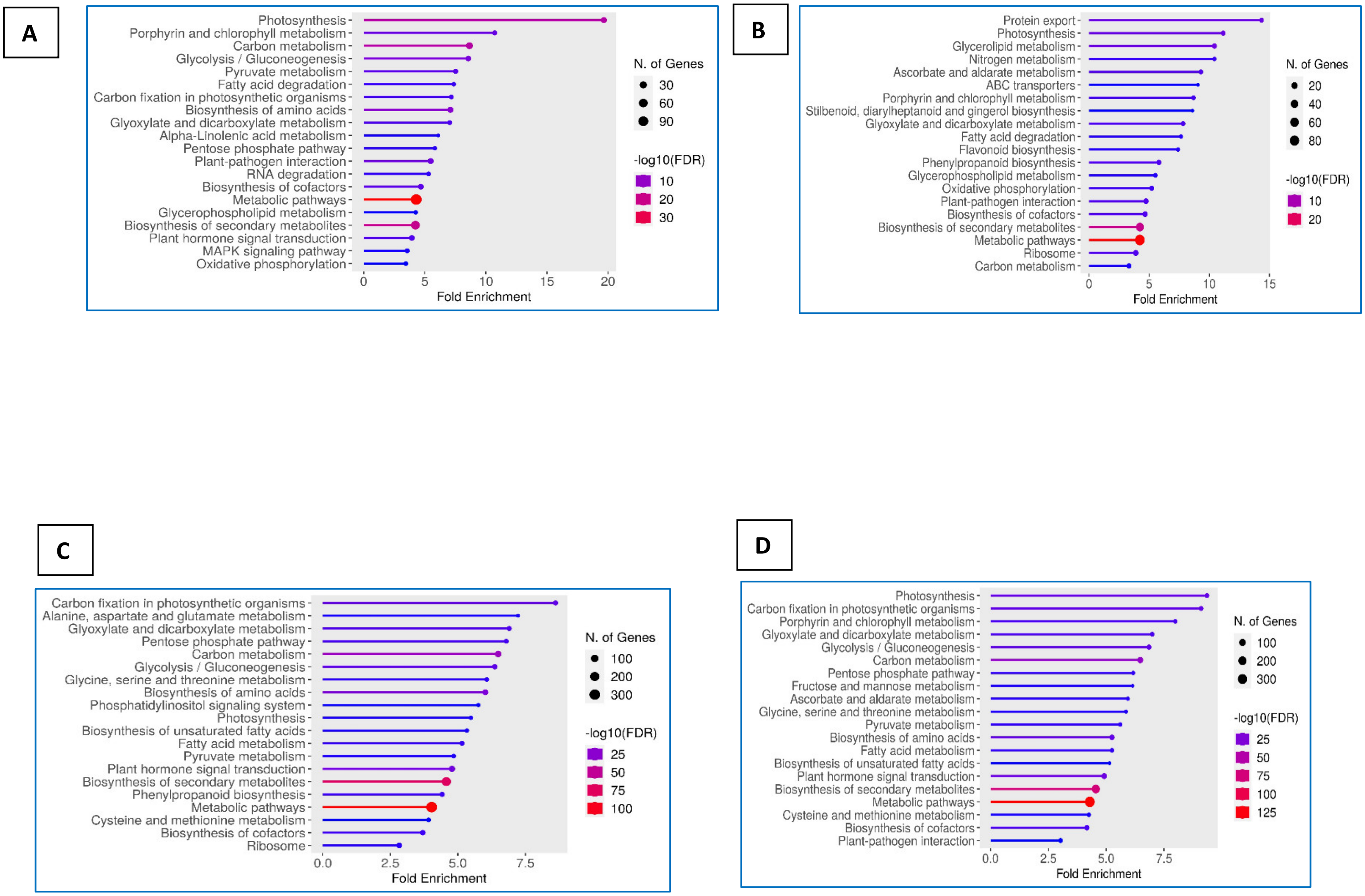

Supplementary Figure S5: Top 20 KEGG pathways enriched in different tissues A) Root tissue B) FPL Tissue] and among cultivars [C) Western tissue D) Eastern cv drawn in shinyGO0.77 using *Daucus carota* subsp *sativus* as a reference

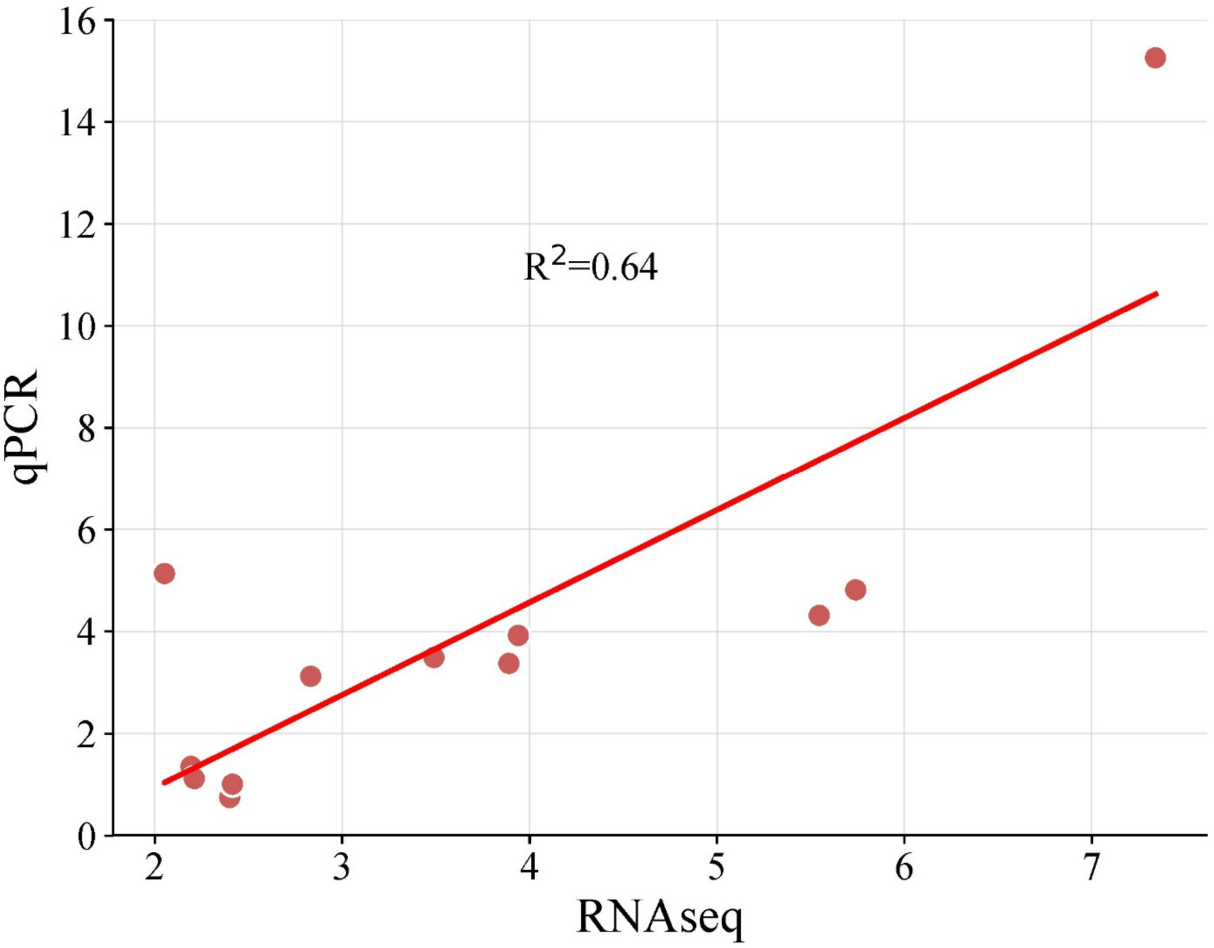

Supplementary Figure S6: **Validation of DEGs identified in RNA seq by quantitative real-time PCR (qRT-PCR) based on Log_2FC and linear regression method**
